# Possible Integrative Actions of Leptin and Insulin Signaling in the Hypothalamus Targeting Energy Homeostasis

**DOI:** 10.3389/fendo.2016.00138

**Published:** 2016-10-20

**Authors:** Mina Thon, Toru Hosoi, Koichiro Ozawa

**Affiliations:** ^1^Department of Pharmacotherapy, Graduate School of Biomedical and Health Sciences, Hiroshima University, Hiroshima, Japan

**Keywords:** leptin, insulin, GRP78, leptin resistance, endoplasmic reticulum stress, food intake

## Abstract

Obesity has emerged as one of the most burdensome conditions in modern society. In this context, understanding the mechanisms controlling food intake is critical. At present, the adipocyte-derived hormone leptin and the pancreatic β-cell-derived hormone insulin are considered the principal anorexigenic hormones. Although leptin and insulin signal transduction pathways are distinct, their regulation of body weight maintenance is concerted. Resistance to the central actions of leptin or insulin is linked to the emergence of obesity and diabetes mellitus. A growing body of evidence suggests a convergence of leptin and insulin intracellular signaling at the insulin–receptor–substrate–phosphatidylinositol-3-kinase level. Moreover, numerous factors mediating the pathophysiology of leptin resistance, a hallmark of obesity, such as endoplasmic reticulum stress, protein tyrosine phosphatase 1B, and suppressor of cytokine signaling 3 also contribute to insulin resistance. Recent studies have also indicated that insulin potentiates leptin-induced signaling. Thus, a greater understanding of the overlapping functions of leptin and insulin in the central nervous system is vital to understand the associated physiological and pathophysiological states. This mini-review focuses on the cross talk and integrative signaling of leptin and insulin in the regulation of energy homeostasis in the brain.

## Leptin

**Figure 1:**
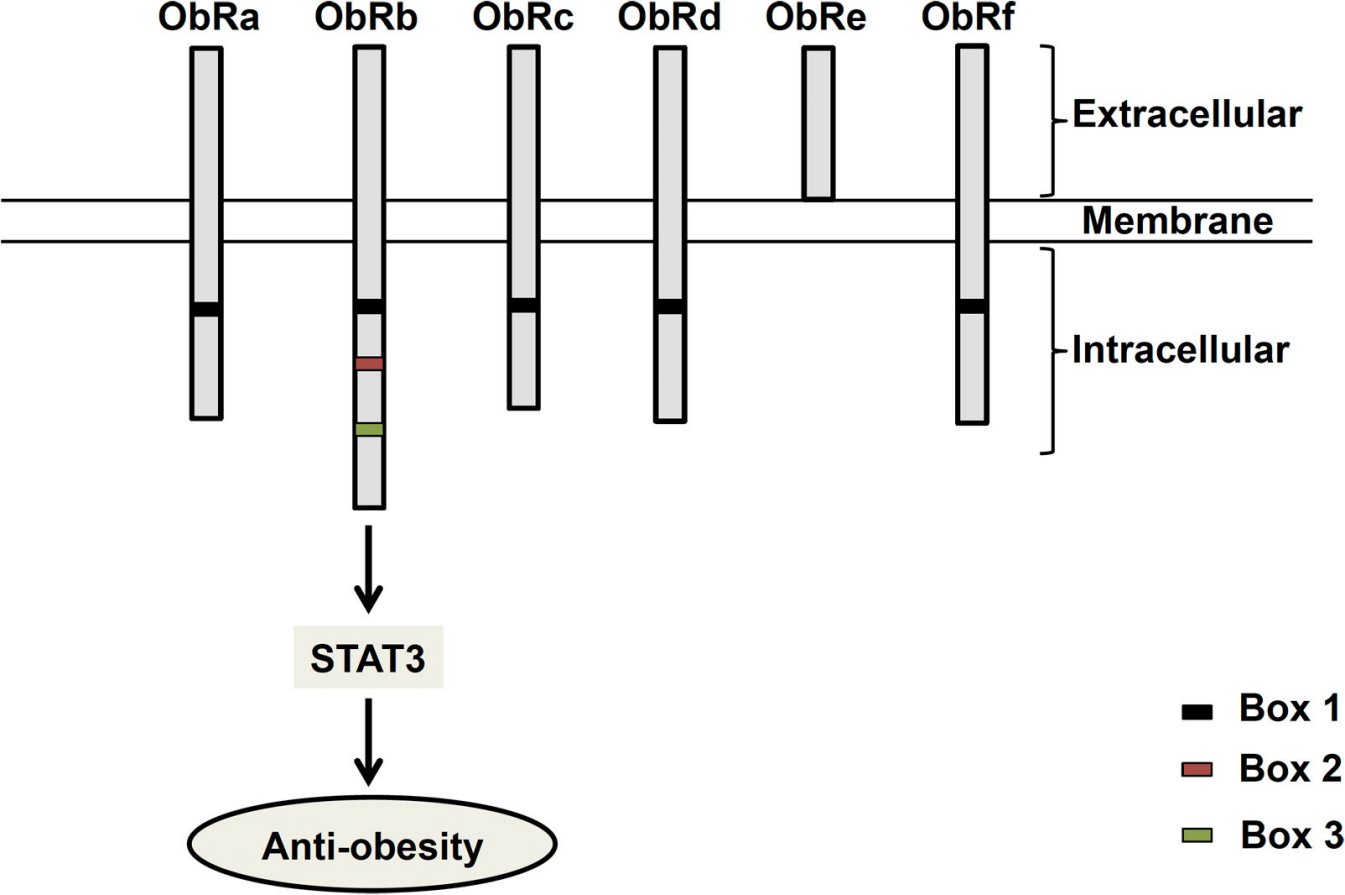
**Structure of leptin receptor isoforms**. Six different spliced isoforms of the leptin receptors (ObR) have been documented as ObRa–ObRf. All the isoforms share identical extracellular binding domain. ObRb possesses the longest intracellular domain, which is important for leptin signaling.

Since its discovery in 1994 (1), the 16 kDa adipocyte-derived hormone leptin has attracted interest in the field of obesity research due to its role in the regulation of energy balance (1–3). Leptin, secreted from adipose tissues in proportion to the fat store (4), acts in the hypothalamus to regulate feeding behavior (1–3). Leptin receptors (ObR) exist as six different isoforms (ObRa–ObRf), classified as short (ObRa, ObRc, OBRd, and ObRf), long (ObRb), and secreted (ObRe) isoforms (5, 6) (Figure 1). ObRa, ObRb, ObRc, ObRd, and ObRe were reported to be expressed in mice (6) and ObRa, ObRb, ObRc, ObRf (7), and ObRe (8) in rats. In humans, expression of ObRa, ObRb, ObRc (9), and ObRe (10) has been described. ObRa, ObRb, ObRc, ObRd, and ObRf isoforms are transmembrane receptors that share the characteristic of possessing a box 1 motif-binding Janus kinase 2 (JAK2). ObRe is the only isoform of the leptin receptor lacking a transmembrane domain (10–12). ObRb, the only isoform featuring a full-length intracellular domain for interaction with other proteins functioning as intracellular signal transducers, is considered the main functional receptor of leptin (13, 14), while the roles of the other isoforms remain to be elucidated (10, 15). ObRb expression is very high in the hypothalamus, where its role is well known in mediating body weight regulation. ObRb is a member of the class I cytokine receptor family, which mediates the Janus kinase signal transducer and activator of transcription (JAK-STAT) pathway (16). By binding to ObRb, leptin activates multiple signaling cascades, such as JAK2–STAT3 (14), mitogen-activated protein kinase/extracellular signal-regulated kinase (MAPK/ERK) (17, 18), and phosphatidylinositol 3-kinase/protein kinase B (PI3K/Akt) pathways (19). The major anti-obesity effects of leptin are initiated by the phosphorylation of JAK2. The activation of JAK2 permits STAT3 phosphorylation and nuclear translocation. Nuclear phospho-STAT3 acts as a transcription factor for STAT3-targeted genes (20, 21).

Although leptin has been considered a candidate for combating obesity, leptin insensitivity represents the barrier to its proper function in obese subjects. Consequently, identifying the mechanisms by which leptin resistance develops is critical. Impairment of leptin signaling is thought to be one such mechanism. Suppressor of cytokine signaling 3 (SOCS3), a mediator of negative feedback to STAT3, is known to attenuate leptin-induced signaling, hence SOCS3-deficient mice displayed increased leptin-induced STAT3 phosphorylation in the hypothalamus (22). Similarly, protein tyrosine phosphatase 1B (PTP1B) dephosphorylates JAK2, thereby inhibiting leptin activity. Consistent with this action, deactivation of PTP1B results in a decrease in body weight and adiposity, and an increase in energy expenditure in mice (23). Thus, SOCS3 and PTP1B are molecular mediators of leptin resistance.

## Insulin

The pancreatic hormone insulin is widely known to reduce blood glucose levels *via* stimulation of glucose uptake by peripheral tissues, such as fat, the liver, and skeletal muscle. Insulin signaling is initiated through its binding with and mediation of protein kinase activity in the beta subunit of the insulin receptor (IR) (24). This stimulation permits phosphorylation of the insulin receptor substrate (IRS) to promote the activation of the PI3K–Akt pathway, which is a major metabolic pathway of insulin (25).

In addition to its peripheral actions, insulin enters the brain from the circulation (26). Insulin in the central nervous system (CNS) affects feeding behavior and energy homeostasis (27–29). Several entry pathways of peripheral insulin into the brain have been reported (30, 31). These include the transport of insulin by brain micovascular endothelial cells from peripheral vessels and the delivery of insulin to cerebrospinal fluid (CSF) *via* choroid plexus (30–33). *In vivo* studies have shown the injection of insulin (34) or an insulin-mimetic compound (35) intracerebroventricularly (icv) to reduce food intake in rats. In a similar way of its expression in periphery, IR is expressed in the brain (36). The hypothalamic signaling pathway of insulin activates IRS–PI3K, resulting in the activation of its downstream target protein Akt. Insulin-induced Akt activation elicits Akt’s phosphorylation of the transcription factor forkhead box protein 1 (FoxO1) to suppress the expression of orexigenic neuropeptides (37). This insulin-activated PI3K–Akt pathway may be linked to anorexia, as the administration of PI3K inhibitors has been shown to hinder the effect of insulin on lowering food intake (38).

## The Mechanisms of Leptin and Insulin Resistance

A number of mechanisms have been proposed to explain leptin and insulin resistance. These include alteration of leptin and insulin transport across the blood–brain barrier (BBB) (39, 40), alteration of their intracellular signal transduction [e.g., SOCS3, PTP1B, and endoplasmic reticulum (ER) stress] (22, 23, 41–45), and other such abnormalities. In this part, we will focus on the mechanisms-mediated disruption of leptin and insulin signal transduction.

Endoplasmic reticulum stress is one of the mechanisms involved in defective action of leptin and insulin signaling. The ER, an organelle fulfilling diverse cellular functions, plays critical roles in the folding and quality control of proteins. Accumulation of unfolded or misfolded proteins in the ER disrupts ER homeostasis, which in turn causes ER stress. In reaction to this ER stress, cells trigger an adaptive response termed the “unfolded protein response” (UPR). To restore normalcy in ER function, UPR serves to downregulate protein translation, upregulate several chaperone proteins, and activate degradation pathways to clear the unfolded or misfolded protein from the ER (46–49). ER stress is implicated in a wide range of diseases, including metabolic diseases (50), neurodegenerative diseases (51), and cancers (52). Obesity is associated with the activation of inflammatory pathways and stress response signaling. In the obesity model, fat secretes various cytokines (53) and free fatty acids (FFAs) (54). These factors were suggested to cause ER stress (55). Supporting this theory, the pro-inflammatory cytokines TNFα (56), IL-1β (57), and interferon-γ (58) were shown to induce ER stress. Current evidence suggests that overnutrition may contribute to the development of ER stress and the activation of the UPR signaling pathway (59, 60). For instance, excess dietary saturated fatty acids (SFAs) consumption induces ER stress markers (61). Sensitivity to leptin and insulin is reduced in obese rats (62, 63). Others and we have reported that ER stress is an underlying mechanism mediating leptin resistance (43–45). Insulin resistance is also a hallmark of obesity and type 2 diabetes, and ER stress is known to induce insulin resistance by impairing IR signaling (64). Furthermore, ER stress is known to induce beta cell death, consequently, compromising insulin release (60). ER stress may therefore play a key role in leptin and insulin resistance.

Another mediator implicated in the attenuation of leptin and insulin signaling is PTP1B. PTP1B is involved in the negative regulation of both leptin and insulin signaling (65, 66). PTP1B inhibits leptin and insulin activities *via* dephosphorylation of JAK2 (67) and the activated insulin receptor (42), respectively. In this regard, PTP1B knockout mice increases sensitivity to leptin and insulin, and are resistant to a high-fat diet-induced obesity (42, 68). Therefore, development of potent and specific inhibitors for PTP1B has become interest in the treatment of type 2 diabetes and obesity (69). Over the last decades, diverse PTP1B inhibitors have been developed (70–72).

Besides PTP1B, SOCS3 is another negative regulator of leptin and insulin signaling. SOCS3 inhibits leptin- and insulin-induced signal transduction (41, 73, 74). Deletion of SOCS3 in hypothalamic neurons enhances leptin sensitivity, reduces appetite, and protects from diet-induced obesity (75); while overexpression of SOCS3 in proopiomelanocortin (POMC) neurons leads to hyperphagia and obesity (76, 77). In addition, SOCS3 knockout mice show improvement in glucose tolerance and insulin sensitivity (78). Based on these evidences, molecules that intervene SOCS3 actions would represent a potential therapeutic target in the treatment of obesity and type 2 diabetes.

## Possible Interaction of Leptin and Insulin Activity in the CNS

Leptin and insulin induce the JAK–STAT3 and PI3K–Akt pathways, respectively, in hypothalamic neurons. Although leptin and insulin mediate distinct and common signaling pathways, they are both documented as major regulators of energy homeostasis and adiposity. It has been indicated that the disruption of IRs in the brain may cause obesity, insulin resistance, hyperphagia, and hyperleptinemia in mice (79). Moreover, an increase in food intake and obesity was observed in IRS-2-deficient mice despite their high circulating levels of leptin (80). These findings highlight the probable role of insulin in the control of food intake.

**Figure 2:**
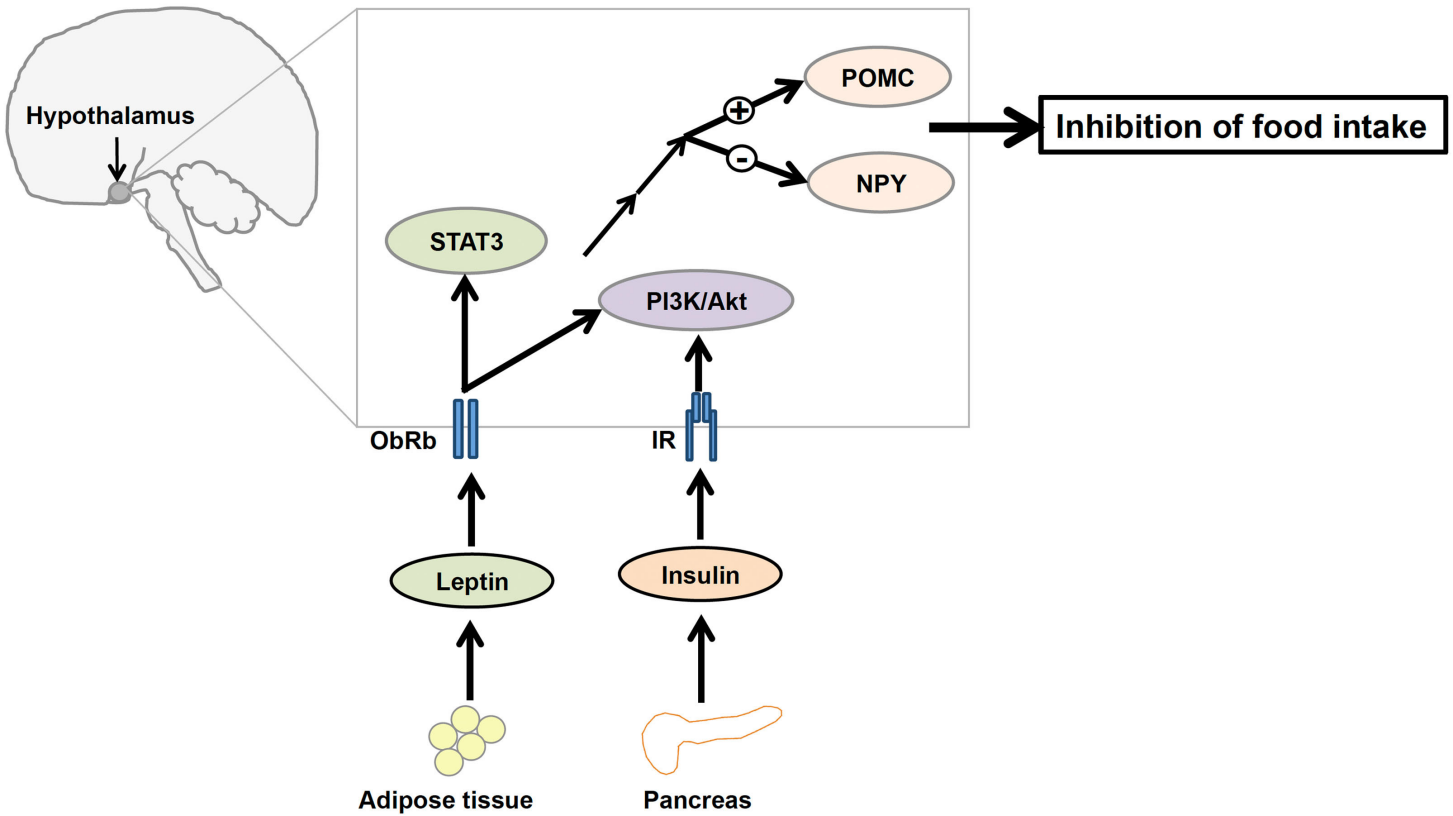
**A diagrammatic representation of the integration of leptin and insulin signals in the regulation of energy homeostasis in the central nervous system**. Adipocyte-derived hormone leptin and pancreatic hormone insulin bind to leptin receptors (ObRb) and insulin receptors (IRs), respectively, in the hypothalamus. Leptin and insulin regulate the expression of proopiomelanocortin (POMC) and neuropeptide Y (NPY) neurons in the hypothalamus *via* the phosphatidylinositol 3-kinase/protein kinase B (PI3K/Akt) signaling pathway to suppress food intake.

Leptin and insulin receptors (IRs) expressed in the CNS mediate the anorexigenic effects of these hormones (81–83). The hypothalamus is the principal site where leptin and insulin exert their regulatory effects on the maintenance of energy homeostasis (Figure 2). Leptin and insulin suppress the activity of the orexigenic neuropeptide Y (NPY)/agouti-related protein (AgRP) neurons, while they stimulate the anorexigenic POMC/cocaine- and amphetamine-related transcript (CART) neurons (84, 85) (Figure 2). Additionally, recent studies have elucidated the effects of leptin and insulin on each other’s actions in the body. For instance, leptin resistance can lead to the inhibition of insulin signaling, while insulin resistance can alter leptin signaling in a hypothalamic cell line (86). Conversely, our study revealed that insulin can potentiate leptin-induced STAT3, a transcription factor critical to a major signaling pathway exerting anti-obesity effects of leptin (87). Thus, it is possible that leptin and insulin may act synergistically to reduce body weight and food intake. If this is the case, cross talk between leptin and insulin would be crucial to the regulation of whole body energy homeostasis.

ObRb and IR share an aspect of their signaling pathways, namely the involvement of PI3K, suggesting that perhaps the IRS–PI3K interaction is a mechanism by which the regulatory effects of both leptin and insulin on the reduction of food intake are integrated (88, 89) (Figure 2). Although the role of leptin-induced PI3K signaling is not well understood, evidence suggests that icv infusion of a PI3K inhibitor interferes with leptin-mediated reduction of food intake (19). Therefore, understanding this molecular mediator that links leptin and insulin signaling in the hypothalamus may be critical to understanding the regulation of energy homeostasis.

Recent literatures have focused on the role of FoxO1 in the regulation of food intake and energy expenditure. FoxO1 stimulates expression of transcription of orexigenic NPY and AgRP, suppresses the transcription of anorexigenic POMC, and blocks STAT3 action in POMC and AgRP neurons. Mechanistically, the activation of PI3K/Akt signaling pathway by insulin leads to phosphorylation of its downstream mediator, FoxO1. Inactivation of FoxO1 by its phosphorylation results in its translocation from the nucleus to cytoplasm, allowing STAT3 to bind to POMC or AgRP promoter (90, 91). IRS/PI3K/Akt axis is crucial for both leptin and insulin in the CNS (38, 92). In addition, the deletion of FoxO1 in POMC neuron results in weight loss and increases leptin sensitivity (93). Based on these evidences, FoxO1 might be a mediator of the potential cross talk between leptin and insulin in the regulation of food intake.

As mentioned above, SOCS3 and PTP1B are molecules that inhibit leptin signaling. Interestingly, SOCS3 and PTP1B also serve as regulators of insulin signaling. It has been suggested that SOCS3 suppresses IR signaling (41). Conversely, mice lacking PTP1B show enhanced insulin sensitivity (42). Therefore, SOCS3 (94) and/or PTP1B (95, 96) appear to share the suppressive effects on leptin and insulin signaling that are commonly seen in obesity.

Endoplasmic reticulum stress contributes to both leptin and insulin resistance in obesity. Thus, interventions that alleviate ER stress, by, for instance, improving protein folding *via* increasing chaperone capacity, would offer a potential therapeutic approach for the amelioration of obesity and ER-stress-related diseases. Recent literature proposes the involvement of 78 kDa glucose-regulated protein (GRP78) in the regulation of whole-body insulin sensitivity (97), glucose homeostasis (98), and protection against ER stress (99). Moreover, 4-phenylbutyrate (4-BPA), a chemical chaperone that enhances protein folding (100), was shown to reverse ER-stress-induced leptin resistance (43, 44). Furthermore, BPA alleviated FFAs-induced insulin resistance and beta cell dysfunction (101). Additionally, ob/ob mice, given a chemical chaperone, showed a reduction in ER stress markers, and improved insulin sensitivity and glucose homeostasis (99). Of note, GRP78 levels can be upregulated by insulin (102) and leptin (103) themselves. Based on a growing body of supporting evidence, it seems possible that GRP78 might be a candidate for therapeutic application, working by contributing to the actions of leptin, and insulin in the maintenance of energy homeostasis. On the other hand, the small GTPase Rap1 in the CNS has recently been identified as a key component in development of high-fat diet-induced obesity through ER stress (104). The suppression of Rap1 protect against obesity and metabolic disorders through the regulation of food intake and maintaining leptin and insulin signaling (104). Thus, manipulation of neuronal Rap1 would represent a potential therapeutic target for obesity.

In conclusion, the central signaling of leptin and insulin may be vital in controlling energy homeostasis *via* feeding suppression. Resistance to the actions of leptin or insulin is associated with the pathophysiology of obesity and type 2 diabetes. An advanced understanding of the physiological and pathophysiological actions of leptin and insulin in the CNS will shed light on potential therapeutic interventions for obesity.

## Author Contributions

MT and TH wrote the manuscript. KO checked the manuscript.

## Conflict of Interest Statement

The authors declare that the research was conducted in the absence of any commercial or financial relationships that could be construed as a potential conflict of interest.
